# Low-Grade Squamous Intraepithelial Lesion in the Setting of Human Papillomavirus and Chlamydia Coinfection

**DOI:** 10.7759/cureus.26254

**Published:** 2022-06-23

**Authors:** Beatriz De Faria, Merna Haridi, Alana Hutcheson, Teja Mehendale

**Affiliations:** 1 Dr. Kiran C. Patel College of Osteopathic Medicine, Nova Southeastern University, Fort Lauderdale, USA; 2 Medical School, St. Martinus University, Willemstad, CUW; 3 Family Medicine, Pontiac General Hospital, Pontiac, USA

**Keywords:** vulvar lesions, genital warts, cervical dysplasia, cervical intraepithelial neoplasia, condyloma acuminata, chlamydia, human papillomavirus (hpv)

## Abstract

Human papillomavirus (HPV) is the most common sexually transmitted infection (STI) in the United States. It most commonly affects the genital areas, as well as the mouth and throat. Research has shown that HPV is a cause of cervical cancer and *Chlamydia trachomatis* is a potential cofactor in the development of cervical intraepithelial neoplasia (CIN). However, there have been limited cases reported on understanding this coinfection and its mechanism through the lens of molecular biology. We present a case of a 22-year-old female with complaints of persistent lesions on the labia for more than 6 months that have increased in number. Histopathology was suggestive of HPV. This case report emphasizes the importance of HPV and chlamydia coinfection as leading causes for persistence of condyloma acuminatum and low-grade squamous intraepithelial lesion (LSIL), and the importance of screening and clinically managing vaginal HPV.

## Introduction

Cervical pathology that exhibits abnormal cervical cells whether precancerous or cancerous are commonly associated with human papillomavirus (HPV). Most commonly, high-risk HPV, such as HPV 16 and HPV 18, have been found to play a role in the transformation of cervical epithelial cells by inhibiting tumor suppressors such as TP53 and pRb [1]. Although HPV plays a huge role in cervical dysplasia and carcinoma, there are other factors and events leading to the development of dysplasia and cancer. One of these factors is the coinfection of *Chlamydia trachomatis (C. trachomatis)* which has the potential to disrupt the cervical epithelium and therefore, increase HPV persistence, and increase the risk of dysplasia and neoplasia [2]. Its role in oncogenesis has been linked to its effect on inhibiting the mismatch repair (MMR) pathway and causing DNA breakage which together can lead to uncontrolled proliferation [3]. This case study focuses on HPV and chlamydia coinfection as a driving factor for the persistence of both condyloma acuminatum and low-grade squamous intraepithelial lesion (LSIL) on a molecular level.

## Case presentation

**Figure 1:**
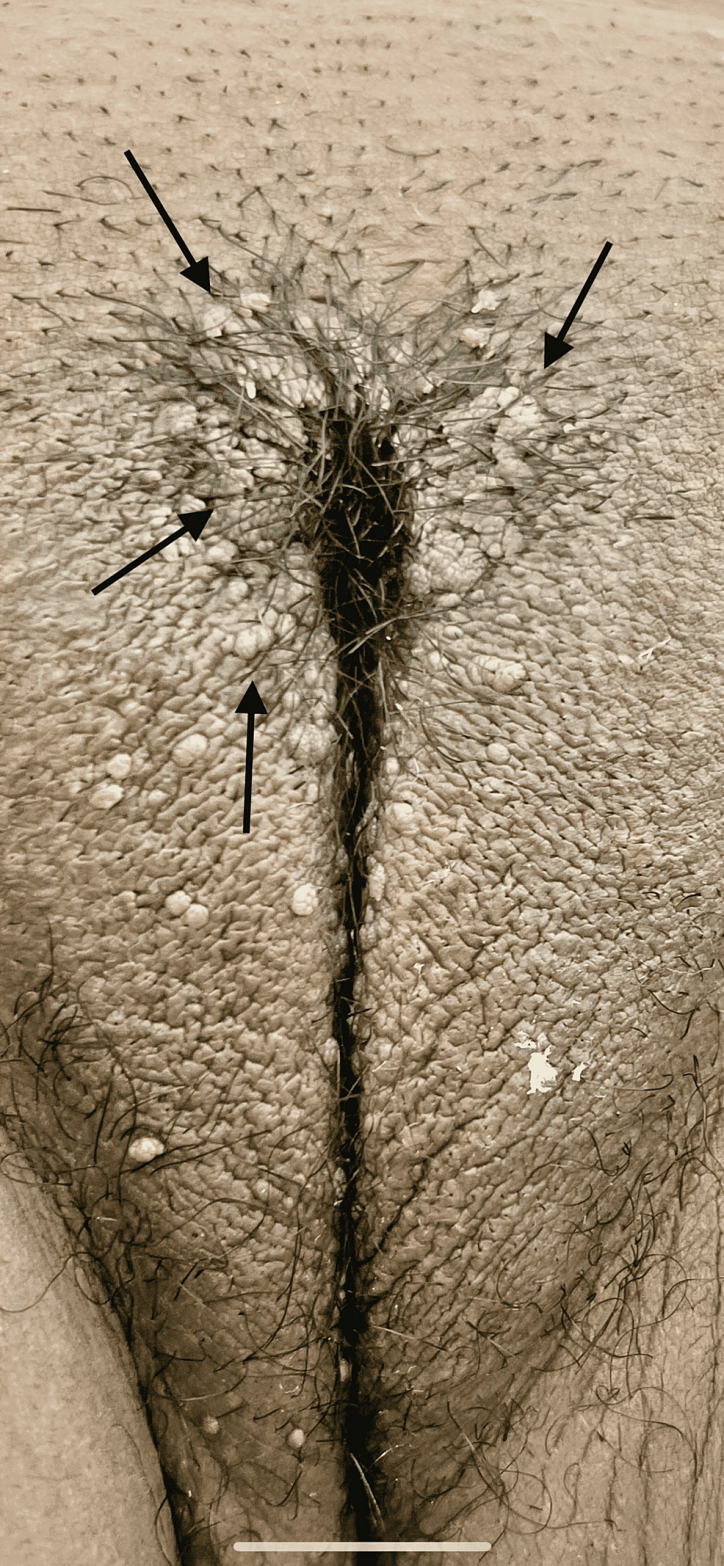
Vulvar lesions before trichloroacetic acid (TCA) treatment Arrows indicate vulvar lesions

A 22-year-old female with a past medical history of polycystic ovarian syndrome (PCOS) presented to the family medicine clinic to establish care with a primary care provider. The patient complained of lesions around her labia that have persisted for at least 6 months. She has never had a cervical exam done before and has never been tested for any sexually transmitted disease in the past. Upon pelvic examination, multiple (>20) 1-2 mm wart-like lesions were located on the labia majora (Figure 1). At that time, the lesions were a tan-brown color similar to the patient’s skin, soft, cauliflower-like, and without any complaints such as pain, redness, or itching. The rest of the physical exam was unremarkable. Papanicolaou (Pap) smear and sexually transmitted illness (STI) screening samples were collected.

The patient was found to be positive for *C. trachomatis* infection and cervical cytology resulted in LSIL. She was promptly treated for *C. trachomatis* with doxycycline. The patient returned to the clinic for a biopsy of the vulvar lesions. Due to the LSIL diagnosis, HPV sample was also collected at that time. The biopsy found that the culprit of the lesions was condyloma acuminatum. E6/E7 viral messenger RNA from 14 high-risk HPV types (16,18,31,33,35,39,45,51,52,56,58,59,66,68) were not detected.

**Figure 2:**
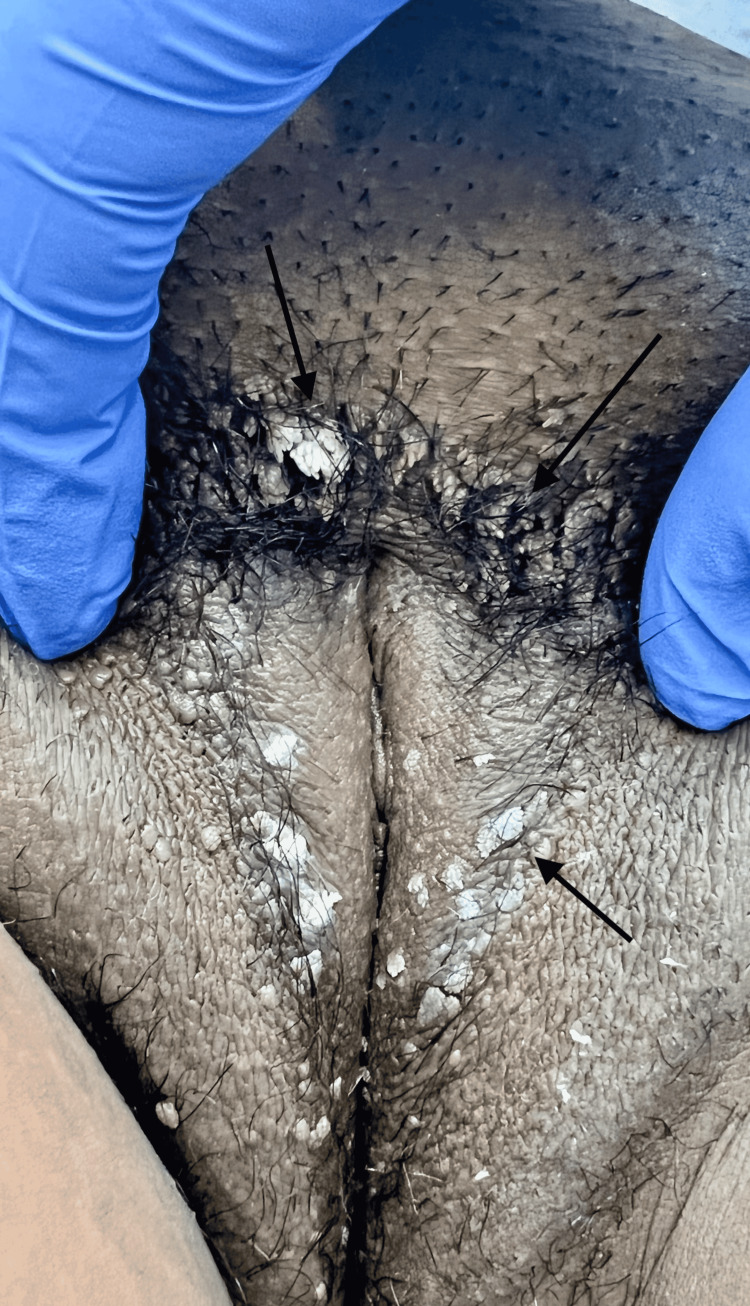
Vulvar lesions immediately after first trichloroacetic acid (TCA) treatment Arrows indicate vulvar lesions after first treatment with trichloroacetic acid

The patient was advised to return for another Pap smear test in 6 months due to LSIL without high-risk HPV finding and to return sooner to discuss trichloroacetic acid (TCA) treatment for venereal warts. When the patient returned, lesions around the labia were diffused bilaterally without improvement in number or appearance; however, the patient was complaining of great discomfort due to itching. She consented to proceed with the TCA treatment (Figure 2). The patient tolerated the first TCA treatment well. After treatment, multiple warts still persisted but five warts of larger size reduced in size. The patient agreed to continue TCA treatment weekly and will follow up in 6 months for a Pap smear.

## Discussion

*C. trachomatis* is a common sexually transmitted illness. Although it can be easily treated, it can often be asymptomatic and lead to damage to the female reproductive tract through the formation of scar tissue [4]. Not only can it cause such permanent damage but it also has the potential to transform cervical epithelium.

*C. trachomatis* affects the MMR pathway by inhibiting the expression of MMR genes [3]. This pathway’s role is to correct any errors after replication such as insertions or substitutions. When there are errors in this pathway, genomic stability cannot be maintained. This leads to microsatellite instability (MSI) which has been associated with many cancers, including cervical cancer [5]. *C. trachomatis*’ inhibition of MMR is via proteasomal degradation of E2F1, a transcription factor. Such inhibition of this pathway is thought to promote mutation and therefore oncogenesis [3].

HPV’s role in cervical pathology is widely known and it has played a role in current cervical screening practices. The recommendations set by the American College of Obstetricians and Gynecologists (ACOG) are to start screening using cytology alone from ages 21 to 29. From ages 30-65 one can either get cytology alone every 3 years, HPV testing alone every 5 years, or HPV and cytology co-testing every 5 years [6]. Although many HPV infections can resolve on their own, the coinfection with *C. trachomatis* has been found to be a risk factor for HPV persistence [2,7].

However, *C. trachomatis* and other possible coinfections do not currently play a role in screening guidelines for cervical cancer.

Our patient presented with diffuse and persistent vulvar warts that were soon identified as condyloma acuminatum. During further evaluation, our patient was diagnosed with *C. trachomatis*. Although the patient was found to be negative for high-risk HPV types, cervical cytology resulted in LSIL.

## Conclusions

This case illustrates the role that *C. trachomatis* plays in cellular reprogramming. At a young age, it is expected that HPV infection will resolve on its own and be of low risk for disease. Yet, this case demonstrated that when chlamydia coinfection is involved, the patient’s HPV-related disease, even without detection of high-risk HPV, led to persistent and numerous warts as well as a diagnosis of LSIL. Although the role of chlamydia in cellular reprogramming continues to be studied, this case supports current studies that such coinfection plays a role in disease. This should prompt clinicians to screen for chlamydia frequently in order to treat it before it can play a role in cellular reprogramming, and further the risk for cervical and vulvar disease. Although it would be prudent to observe and screen patients with current or past history of *C. trachomatis* for cervical cancer more carefully, more studies need to continue to be done before outlining new preventive guidelines.

## References

[1] Burd EM (2003). Human papillomavirus and cervical cancer. Clin Microbiol Rev.

[2] Mosmann JP, Zayas S, Kiguen AX, Venezuela RF, Rosato O, Cuffini CG (2021). Human papillomavirus and Chlamydia trachomatis in oral and genital mucosa of women with normal and abnormal cervical cytology. BMC Infect Dis.

[3] Koster S, Gurumurthy RK, Kumar N (2022). Modelling Chlamydia and HPV co-infection in patient-derived ectocervix organoids reveals distinct cellular reprogramming. Nat Commun.

[4] Cates W Jr, Wasserheit JN (1991). Genital chlamydial infections: epidemiology and reproductive sequelae. Am J Obstet Gynecol.

[5] Li G-M (2008). Mechanisms and functions of DNA mismatch repair. Cell Res.

[6] US Preventive Services Task Force (2018). Screening for cervical cancer: US preventive services task force recommendation statement. JAMA.

[7] Silins I, Ryd W, Strand A (2005). Chlamydia trachomatis infection and persistence of human papillomavirus. Int J Cancer.

